# Infectious Disease Control and Management in Ethiopia: A Case Study of Cholera

**DOI:** 10.3389/fpubh.2022.870276

**Published:** 2022-05-30

**Authors:** Se Eun Park, Yeonji Jeon, Sunjoo Kang, Abel Gedefaw, Dejene Hailu, Biruk Yeshitela, Moti Edosa, Mesfin Wossen Getaneh, Mekonnen Teferi

**Affiliations:** ^1^Clinical, Assessment, Regulatory, Evaluation (CARE) Unit, International Vaccine Institute, Seoul, South Korea; ^2^Yonsei University Graduate School of Public Health, Seoul, South Korea; ^3^College of Medicine and Health Sciences, Hawassa University, Hawassa, Ethiopia; ^4^School of Public Health, Hawassa University, Hawassa, Ethiopia; ^5^Bacterial and Viral Disease Research Directorate, Armauer Hansen Research Institute, Addis Ababa, Ethiopia; ^6^Diseases Surveillance and Response Directorate, Ethiopian Public Health Institute, Addis Ababa, Ethiopia; ^7^Clinical Trials Directorate, Armauer Hansen Research Institute, Addis Ababa, Ethiopia

**Keywords:** cholera, OCV, national cholera control plan, case detection, case management, outbreak response, health system, Ethiopia

## Abstract

Cholera remains a significant public health problem among the vulnerable populations living in many resource-limited settings with poor access to safe and clean water and hygiene practice. Around 2.86 million cholera cases and 95,000 deaths are estimated to occur in endemic countries. In Ethiopia, cholera has been one of the major epidemic diseases since 1634 when the first cholera outbreak was recorded in-country. Several cholera epidemics occurred with recent outbreaks in 2019–2021. Cholera has been often reported as acute watery diarrhea due to limited diagnostic capacity in remote areas in Ethiopia and sensitivities around cholera outbreaks. The government of Ethiopia has been executing several phases of multi-year health sector development plan in the past decades and has recently developed a national cholera control plan. Here, we aim to present the existing cholera control guidelines and health system in Ethiopia, including case detection and reporting, outbreak declaration, case management, and transmission control. Challenges and way forward on further research and public health interventions are also discussed to address the knowledge and health service gaps related to cholera control in Ethiopia.

## Introduction

Cholera is a diarrheal disease caused by the gram-negative bacteria *Vibrio cholerae (V. cholerae)* infection that can cause extreme loss of fluid and severe dehydration. The disease remains a significant public health problem for people with poor access to safe and clean water, sanitation, and hygiene (WaSH) practice; a proxy indicator of a country's lagging socio-economic development. Around 2.86 million cholera cases and 95,000 deaths are estimated to occur in endemic countries in 2015 (1–3). Of the countries with over 1,000 cholera deaths annually, all are in Africa except for India, Bangladesh, Haiti, and Sudan (1, 4). According to the recent Global Burden of Disease (GBD) Study, diarrhea was the eighth leading cause of death among all ages (1.66 million deaths, 95% uncertainty interval) in 195 countries worldwide in 2016; overall diarrheal mortality was 22.4 deaths per 100,000 with higher rates among children younger than 5 years (70.6 deaths per 100,000) (5). *V. cholerae* has been the third leading cause of diarrhea mortality among all ages, responsible for 107,290 deaths, which included 52,232 deaths among children <5 years of age (5). Further, approximately 750,000 of a total of 4.3 million deaths of African children up to 4 years of age are reportedly associated with diarrheal diseases (6). A recent analysis on childhood diarrheal morbidity and mortality in Africa during 2010 and 2015 exhibited diarrheal diseases as the third leading cause of disease and death in children younger than 5 years of age, responsible for an estimated 30 million cases of severe diarrhea and 330,000 deaths in 2015 (7).

As the SARS-CoV-2 (COVID-19) pandemic hit the world since the beginning of 2020, cholera epidemics coexisted with the pandemic. The number of cholera cases reported to the World Health Organization (WHO) has dropped significantly in 2020; with reports of 323,320 cholera cases and 857 deaths in 27 countries among 80 countries that reported cholera, 65% decrease compared to 2019 whereby 923,037 cases and 1,911 deaths were reported globally (8). This phenomenon can be explained in several aspects. The COVID-19 pandemic has also put increased pressure on existing health systems in cholera-endemic countries (4). It is highly likely that the national and local public health surveillance system and laboratory diagnostic capacities for cholera detection and reporting may have been over-stretched, as existing healthcare personnel and resources are prioritized for COVID-19 surveillance and pandemic control (8). The promotion of personal hygiene and hand washing, social distancing, and even lock down measures in some countries during the COVID-19 pandemic may have had some impact on the cholera transmission dynamics (8). Albeit the overall decrease in the number of global cholera cases reported during the first year of COVID-19 pandemic in 2020, Ethiopia has been reporting cholera epidemics in 2019 and throughout 2020 and 2021 (8, 9).

In Ethiopia, cholera has been one of the major epidemic diseases for centuries. Since 1634, when the first cholera outbreak was recorded in Ethiopia with the name of *fangal* (subsequently used for cholera) (10), existing studies suggest that at least five cholera epidemics followed in Ethiopia in the 19^th^ and early 20^th^ centuries, including several outbreaks in more than one waves; cholera outbreaks between 1831–1836 (two separate waves), 1856 and 1866–1867, during the great famine in 1889–1892 (two waves), and 1906 (10, 11) Since 1970, when the seventh global cholera pandemic driven largely by the O1 serogroup and the El Tor biotype reached Africa (12), cholera cases have been subsequently reported in Ethiopia. A comprehensive phylogenetic analysis of contemporaneous African *V. cholerae* strains between 1966 and 2014 exhibited past cholera epidemics in Africa attributable to a single expanded lineage introduced at least 11 times since 1970 (12). The two El Tor strains were introduced to Africa in 1970 with serotype Ogawa in West Africa and serotype Inaba in East Africa particularly in Ethiopia (12). The 1970 *V. cholerae* isolates from Ethiopia are likely associated with importation from the Middle East, and the subsequent cholera epidemics in the continent linked to the multidrug resistant (MDR) sublineages from parts of Asia since 2000 (12) More recent publications on cholera outbreaks in Ethiopia elaborated frequent cholera outbreaks in parts of Ethiopia associated with the 1985–1986 epidemic in the Horn of Africa; Ogawa strain in Ethiopia caused or linked to outbreak in Northern Somalia (13, 14). Cholera re-emerged in Ethiopia in 1993 that affected both urban and rural areas of regional states of Oromiya and Somali and the Addis Ababa, and subsequently in 1994 albeit irregular institutional reporting of cholera cases (15). No extensive recurrence of cholera was reported in 1996 and 1997, but an epidemic reappeared in 1998 and in the 2000s; case series in 2004 and large outbreak in 2006 (15). Reports of cholera cases in the 1990s and 2000s remained irregular in Ethiopia and often reported as acute watery diarrhea (AWD) (16), but cases have been reported continuously with most recent cholera epidemic declarations by the Ethiopian government in 2019, 2020 and 2021 (17).

In response to these past cholera outbreaks in Ethiopia, the government has been taking measures for cholera outbreak investigation and control, setting-up of cholera treatment centers and case management. In 2019, the Ethiopian government has also requested the World Health Organization (WHO) Oral Cholera Vaccine (OCV) International Coordinating Group (ICG) for the emergency use of OCVs, and utilized the bilateral diplomatic channel to get support from the government of Republic of Korea with OCV doses for large-scale reactive mass vaccination campaigns to control cholera outbreaks. More recently, the government of Ethiopia has also officially expressed the commitment for national cholera elimination roadmap and developed a comprehensive multi-sectoral national cholera control plan (NCP) (18). This multi-year government plan entitled the “Multi-sectorial Cholera Elimination Plan, Ethiopia 2021–2028” has been developed (18) in alignment with the WHO Global Task Force for Cholera Control (GTFCC) ‘Ending Cholera – Global Roadmap to 2030' (19), and submitted to the WHO GTFCC in 2021 for endorsement.

Here, we aimed to review the existing cholera control guidelines in Ethiopia, which includes: Section 1 on cholera surveillance and outbreak investigation covering cholera case detection and reporting and outbreak investigation; Section 2 on cholera outbreak control and management, focused on responsibilities of government stakeholders at different levels and health facilities, outbreak response, case management, transmission control, and use of OCV; Section 3 on the Ethiopian government's commitment for cholera control and elimination in Ethiopia; and Section 4 on challenges and way forward to enhance cholera control and prevention in the country. Our intended audiences are national public health leaders and managers, policy makers in health and finance ministries, public health professionals involved in sentinel-based surveillance and community engagement for early cholera case detection, outbreak preparedness and effective outbreak controls, and other national and global stakeholders in bilateral and multilateral aids or technical supports.

## Section 1: Cholera Surveillance and Outbreak Investigation in Ethiopia

### Cholera Case Detection and Reporting

The Guideline on Cholera Outbreak Management in Ethiopia published by Ethiopian Public Health Institute [EPHI; former Ethiopia Health and Nutrition Research Institute (EHNRI)] states cholera as a mandatory notifiable disease (20). All suspected cholera cases need to be reported upon identification. Suspected case is defined as any person 5 years of age or more with profuse AWD and vomiting. Confirmed case of cholera, defined as suspected case with *V. cholerae* O1 or O139 isolated from stool, is sufficient for an outbreak to be declared (20) More specifically, cholera outbreak declarations are made: in cholera epidemic areas, when a patient aged 5 years or more who develops AWD, with or without vomiting is detected; and in an area where cholera is not known to be present, when a patient aged 5 years or more who develops severe dehydration or dies from AWD is detected (20). This implies the importance of existing public health disease surveillance system and reporting capacity. Cholera case detection and outbreak declaration is also closely associated with the healthcare seeking behaviour of local populations and cholera rapid diagnostics and laboratory confirmation ability. In Ethiopia, there are 16 public health laboratories nation-wide; EPHI National Reference Laboratory located in Addis Ababa, Armauer Hansen Research Institute (AHRI) laboratory in Addis Ababa for handling medical research samples, and 14 Regional Laboratories across the country (Table 1).

**Table 1:** National public health laboratories in Ethiopia.

**Laboratories^**1**^**	**Lab type**	**BSL^**2**^**	**Lab location**	**Areas covered**	**Population of areas covered^**3**^**	**Cholera case diagnosis experience**	**Cholera case diagnosis capacity**
Ethiopian Public Health Institute (EPHI) National Reference Laboratory^4^	National Reference laboratory	BSL 2	Addis Ababa (Capital city)	Nation-wide	103 million	Yes	RDT culture serotyping (PCR)
Armauer Hansen Research Institute (AHRI) Laboratory^5^	National Clinical Research Reference laboratory	BSL 2	Addis Ababa (Capital city)	Nation-wide	103 million	Yes	RDT culture serotyping (PCR)
Addis Ababa Regional Laboratory	Regional laboratory	BSL 2	Addis Ababa	Addis Ababa	3.8 million	Yes	RDT culture
Adama Public Health Research & Referral Laboratory	Regional Laboratory	BSL 2	Adama zone	Oromia region	39.0 million	Yes	RDT culture
Afar Public Health Institute Laboratory	Regional laboratory	BSL 2	Semera	Afar region	1.9 million	Yes	RDT culture
Amhara Public Health Institute Laboratory	Regional laboratory	BSL 2	Amhara Bahir Dar	Amahara region	22.5 million	Yes	RDT culture
Amhara Public Health Institute Laboratory - Dessie Branch	Regional laboratory	BSL 2	Dessie	Amhara region (South Wello zone North Wello zone North Shewa zone Oromo Special zone Waghimra-zone)	8.2 million	Yes	RDT culture
Benishangul Gumuz Regional Laboratory	Regional laboratory	BSL 2	Assossa	Benishangul-Gumuz region	1.2 million	Yes	RDT culture
Diredawa Regional Laboratory	Regional laboratory	BSL 2	Dire Dawa	Dire Dawa city	521,000	Yes	RDT culture
Gambella Regional Laboratory	Regional laboratory	BSL 2	Gambella	Gambella region	492,002	Yes	RDT culture
Harari Regional Laboratory	Regional laboratory	BSL 2	Harar	Harar region	270,000	Yes	RDT culture
Nekemte Public Health Research and Referral Laboratory	Regional laboratory	BSL 2	Nekemte, Oromia Regional State	East Wollega West Wollega Horo Gudru Wollega Kelem Wollega Iluabbabor Bunno Bedele Jimma Zone West Shewa zone Jimma town Nekemt town Ambo town	11 million	Yes	RDT culture
Shashemene Public Health Research and Referral Laboratory	Regional Laboratory	BSL 2	Shashemene	West Arsi zone Borena Guiji West Guji Shashemene town Bishan guracha town	7 million	No	None
Somali Regional Laboratory	Regional laboratory	BSL 2	Jigjiga	Somali region	6.4 million	Yes	RDT culture
Southern Nations, Nationalities, and Peoples' Region (SNNPR): Regional State Public Health Laboratory	Regional laboratory	BSL 2	Hawassa	SNNPR region Sidama region	21.0 million	Yes	RDT culture
Tigray Health Research Institute Laboratory	Regional laboratory	BSL 2	Mekele	Tigray region	5.6 million	Yes	RDT culture

1 *The list of national public health laboratories compiled by the EPHI and AHRI and also referred to the Ethiopian National Accreditation Office (ENAO) official webpage*;

2 *BSL, Biosafety level*;

3 *Ethiopia Population Projection Wereda as of July 2021 | Central Statistics Agency official webpage (http://www.statsethiopia.gov.et/population-projection)*;

4 *EPHI laboratory, Established in 1996 as the Ethiopian Health and Nutrition Research Institute (EHNRI) laboratory, and the name changed in 2013 as the EPHI laboratory. Mandated to increase and maintain quality assurance of public laboratories; enhance and implement quality management system of public laboratories; and strengthen laboratory capacity for referral and back-up testing services*;

5 *AHRI laboratory, Mandated to foster evidence-based decision making; improve medical research capacity; foster health innovation and technology transfer; promote local and international participatory research; and improve efficiency of system and ensure accountability*.

### Outbreak Investigation

When a suspected cholera case is reported, a multidisciplinary outbreak investigation team [Rapid Response Team (RRT)] should be organized by the EPHI and an outbreak investigation initiated within 3 hours, according to the EPHI (EHNRI) guideline (20). The RRT is composed of a clinician, lab technician, communication expert, epidemiologist, and environmental health expert (20). The team conducts field assessments to verify reported cholera cases, determine magnitude of cholera outbreak, collect specimens for laboratory confirmation of *V. cholerae*, assess cholera outbreak response capacity at local level, identify high-risk groups, investigate source of contamination, conduct simple on-site control measures, provide emergency treatment supplies, and report findings of outbreak investigation (Table 2) (20). The following variables are to be collected and/or reviewed from the available health facility register: name, age, sex, address, symptoms, date of onset of illness, date treated, treatment provided, treatment outcome (alive, dead, referred), specimen collection status, any risk related data, and index case tracing (20) At community level, interviews of household members and neighbors of cholera cases are to be conducted to assess any recent travel history, contacts with suspected cholera cases or/and ill persons with diarrhea, recent attendance at a funeral (and cause of death of deceased), water sources (drinking, bathing, cleaning kitchen utensils), food consumption history, occupation, and any other risk factors for cholera transmission (20).

**Table 2:** Activities of a Rapid Response Team (RRT) for cholera outbreak investigations.

At health facility	Review/collect data on suspected cholera patients per case definition.
	Review/collect data on patients treated for acute watery diarrhea.
	Assess health facility personnels' understanding on cholera and treatment protocols.
	Make inventory of supplies: specimen collection kits, rehydration supplies, etc.
At community	Interview patients and their families: confirm information on cases, track contracts, and identify risk factors.
	Interview any other ill persons suspected with cholera in the community.
	Interview to assess recent travel history, contacts with suspected cholera cases or/and ill persons with diarrhea, recent attendance at a funeral (and cause of death of deceased), water sources (drinking, bathing, cleaning kitchen utensils), food consumption history, occupation.
Specimens and lab tests	Collect 5–10 rectal swabs (if health facility has not performed) per outbreak/Woreda.
	Do not delay treatment of dehydrated patients to collect specimens.
	Obtain specimens before antibiotic therapy begins.
	Specimen collection within 5 days of onset of illness recommended.
	Arrange transport of rectal swabs to Regional Reference Laboratories and National Reference Laboratory at EHNRI (EPHI).
	Confirm cholera: identify strain, biotype, serotype, antibiotic sensitivity.
Data analysis	Review following information from register: name, age, sex, address, symptoms, date of onset of illness, date treated, treatment provided, treatment outcome (alive, dead, referred), specimen collection status, any risk related data, index case tracing.
	Geographical mapping of cases.
	Graph to visualize daily and accumulated cases per onset of illness.
	Analyse number of cases, deaths, attack rate (AR), case fatality rate (CFR), high risk groups, source of infection, etc.
	Analyze epi-curve to assess if an outbreak is on increase.
	Monitor Weekly Incidence Rate (WIR): i.e., high WIR as a proxy indicator of epidemic and speed of epidemic spread.
Treatment	Ensure treatment of suspected cholera or confirmed cholera patients per treatment guideline.
	Review case management at health facility: i.e., high CFR as a proxy indicator for the need to improve case management.
	Ensure availability of supplies for adequate patient treatment and specimen collection at health facility.
	Set-up a system to provide support for treatment in remotely located communities.
	Provide community health workers with Oral Rehydration Solutions (ORS).
Outbreak control	Conduct on-site control measures to prevent further transmissions linked to any identified source of infection.
	Communicate and sensitize communities and high-risk groups with simple health education messages.
Report and	Report outbreak investigation results and actions taken.
follow-ups	Follow-up surveillance visit(s).

*Reconstructed based on the Guideline on Cholera Outbreak Management Ethiopia, Ethiopia Health and Nutrition Research Institute [EHNRI (now EPHI)], 2011*.

*RRT, Rapid Response Team; EHNRI, Ethiopia Health and Nutrition Research Institute; AR, Attack Rate; CFR, Case Fatality Rate; WIR, Weekly Incidence Rate; ORS, Oral Rehydration Solutions*.

## Section 2: Cholera Outbreak Control and Management in Ethiopia

### Responsibilities at Various Government Levels and Health Facilities

**Figure 1:**
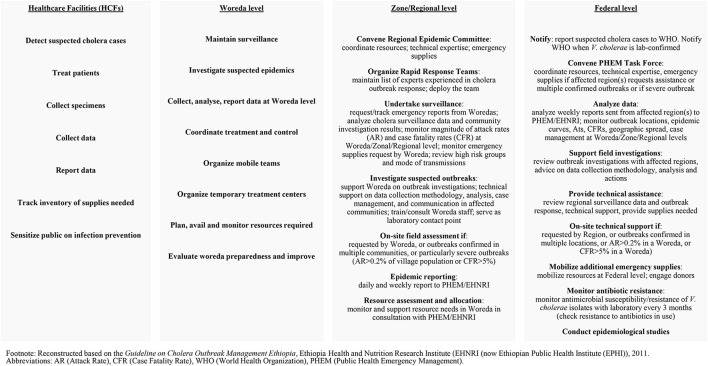
Responsibilities in cholera detection and outbreak control.

Cholera detection and outbreak control include government roles at Woreda, Zone/Regional and Federal levels, responsible for providing adequate and timely support with technical expertise, supplies and resources, situation analysis, decision makings, communications and reporting, etc. (Figure 1). When a cholera outbreak is suspected or confirmed, an Epidemic Control Committee (ECC) must be convened immediately and conduct regular meetings to review responsibilities of stakeholders and track progress in outbreak control (20). The committee members are composed of representatives from multi-sectors and partners for a comprehensive cholera control and prevention approach. Notably, the EPHI (EHNRI) Guideline recommends an inter-country ECC if cholera outbreaks occur near the national border (20). The ECC is also mandated to meet regularly in non-epidemic periods for epidemic preparedness and prevention activities (20).

### Outbreak Response

The key objectives of cholera outbreak response are reducing deaths attributable to cholera and preventing new cholera cases. To achieve these goals, following activities are critical: clear roles and responsibilities at various government and health facility levels, disease surveillance capacity at health facilities, accurate and quick diagnosis at laboratories, quality documentation and reporting, adequate and timely decision-making and allocation of human resources and supplies, proper case management, rapid outbreak investigation and community sensitization to control sources of potential transmission and risk factors, etc. Proper cholera case management requires setting up appropriate Cholera Treatment Centers (CTC) or Cholera Treatment Units (CTU), at bigger and central or smaller and decentralized inpatient facilities, respectively (20). These CTCs or CTUs are typically set-up within existing hospital or hospital compound or health centers or health posts, and aimed at isolating and treating severe cholera patients. For moderate cholera cases, Oral Rehydration Points (ORP) are more widely serviced for early rehydration therapy and quick identification and referral of severe cases to CTC or CTU (20). The EPHI (EHNRI) guideline notes CTC and CTU must function 24 hours while ORP can be open 12 hours/day (20).

### Case Management

Case management begins by assessing clinical conditions of patients visiting health facilities, followed by treatment and discharge (Table 3). The signs of dehydration in patients with AWD is graded based on symptoms reflecting fluid loss (20). Severity of dehydration determines different treatment options; oral rehydration solution (ORS) for moderate dehydration, and intravenous (IV) therapy for severe dehydration (Table 4). Antibiotics may be also used for severely dehydrated patients, after IV rehydration, to reduce volume and duration of diarrhea and period of infectivity. Mass chemoprophylaxis is not recommended but selective chemoprophylaxis by EPHI (20) in alignment with the WHO guideline on cholera control (21). Antibiotics of choice for cholera treatment include doxycycline for adults, amoxicillin syrup for children, and erythromycin for pregnant women (20). For doxycycline resistant *V. cholerae* infection, amoxicillin and erythromycin may be used alternatively. For cholera patients with severe malnutrition, patient's weight loss can be an indicator to confirm dehydration (20). For pediatric cholera and diarrhea, zinc supplementation can be provided to reduce frequency and severity of diarrheal episodes (20). Normal feeding may be resumed when vomiting stops and breastfeeding for infants and young children does not need to be stopped. Cholera patient is eligible for discharge from treatment center when there are no more signs of dehydration and less than three liquid stools in the past 6 hours.

**Table 3:** Flow diagram of cholera case management in Ethiopia.

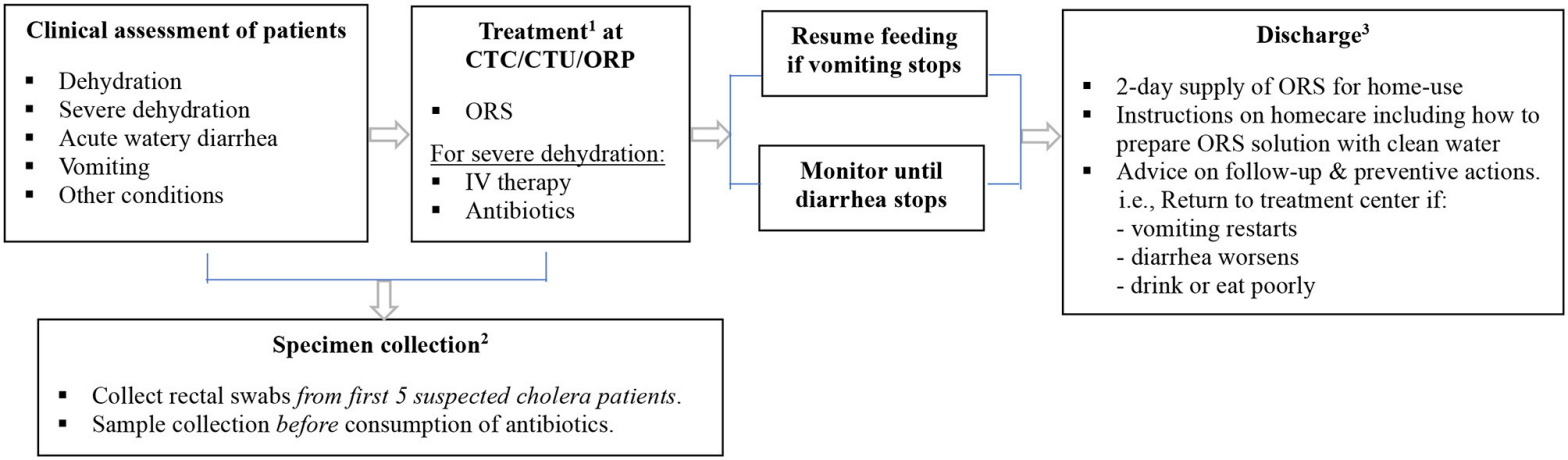

*^1^IV therapy for patients with severe dehydration. ORS during and after IV therapy when patient becomes able to drink. Antibiotics can reduce volume/duration of diarrhea and shorten period of infectivity. Treatment with rehydration of patients should not be delayed by specimen collection. Zinc supplementation for children with watery diarrhea including cholera is also recommended (children aged 6–59 months to receive zinc supplementation for 10 days when vomiting stops). Refer to Table 4 for detailed treatment options for cholera case management*.

*^2^Specimen collection before antibiotics*.

*^3^Discharging patients: For hospitalized patients, transfer to recovery area for continued observation and ORS for 6 h. For patients in recovery area, discharge if no more signs of dehydration and less than three liquid stools in past 6 h*.

*CT, Cholera Treatment Center; CTU, Cholera Treatment Unit; ORP, Oral Rehydration Points; ORS, Oral Rehydration Solutions; IV, Intravenous*.

**Table 4:** Treatment for cholera case management.

		**Acute Watery Diarrhea (AWD) with:**		
		**No dehydration**	**Moderate dehydration** (if two or more signs, including at least one major sign)	**Severe dehydration** (if two or more signs, including at least one major sign)
Signs	Mouth/tongue	Moist	Dry	Very dry
	Thirst^1^	Drinks normally	Thirsty, drinks eagerly^3^	Drinks poorly or not able to drink^3^
	Skin pinch^2^	Goes back quickly	Goes back slowly^3^	Goes back very slowly (>2 sec) ^3^
Treatment^4^		Maintain hydration (Treatment plan A)	ORS (Treatment plan B)	IV, ORS, antibiotic (Treatment plan C)
		• ORS after each loose stool to maintain hydration until diarrhea stops • If patient lives far from treatment center or correct home treatment can't be guaranteed: keep under observation • Sent home with a 2-day supply of ORS with instruction on preparing ORS solution with clean water and schedule: 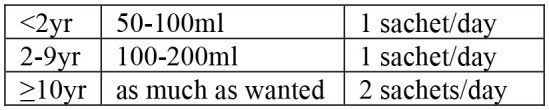 • Instruct patient to return to treatment center if condition deteriorates (if repeated vomiting, number of stools increased, patient drinks or eats poorly) • If patient starts vomiting or develops abdominal distension: Ringer's Lactate 50 ml/kg over 3 h, followed by ORS after assessment of hydration status (monitor every 4 h)	• Admit to treatment center • ORS and monitor until diarrhea/vomiting stops (if patient vomits, wait 10 min, and continue slowly) • Amount of ORS required in 4 h subject to patient's weight (75 ml/kg in 4 h). If patient's weight is unknown, use age 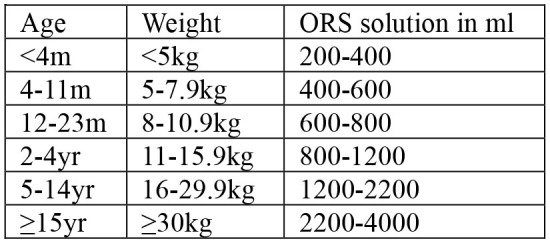 • During first 2 h of treatment: monitor rehydration frequently (at least every hour) • After first 4 hours of treatment: if no more signs of dehydration, follow Treatment Plan A • After first 4 h of treatment: if still signs of moderate dehydration, repeat Treatment plan B for another 4 h and reassess • At any time during treatment: if patient's symptoms deteriorate (if signs of severe dehydration, confused or disorientated, frequent/severe vomiting), immediately shift to Treatment plan C • If patient can't drink and if IV therapy not feasible at treatment center: rehydrate patient using nasogastric tube	• Admit to treatment center • IV treatment immediately to restore normal hydration within 3–6 h: 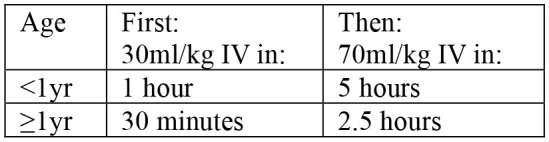 - Ringer's lactate as first choice of IV fluids - If Ringer's lactate not available, normal saline or 5% glucose in normal saline - Plain 5% glucose solution not recommended • If patient can drink, ORS 5 ml/kg/h can be also given simultaneouly with IV drip • If fluid can't be given through IV route, give ORS (20 ml/kg over 6 h) through a nasogastric tube • Assess patient's condition every 30 min during the first 2 h; then every h for next 6–12 h • Monitor pulse and respiratory rates, frequency of urine, stool, and vomiting- Regular urine output (every 3–4 h): good sign of enough fluid- Sign of increasing edema: evidence of over hydration- Sign of continued fast breathing and rapid pulse rate during rehydration: may be early signs of heart failure- Stop rehydration immediately if patient shows any of these signs • Antibiotics^5^ only to patients with severe dehydration to shorten duration of illness and carriage of pathogen

*Reconstructed based on the Guideline on Cholera Outbreak Management Ethiopia, Ethiopia Health and Nutrition Research Institute (EHNRI (now EPHI)), 2011*.

*ORS (Oral Rehydration Solution), IV (Intravenous) rehydration therapy, yr (year), m (month), h (hour)*.

1 *Thirst: Give fluid to patient to observe this sign*.

2 *Skin pinch: Pinch abdominal skin and release to observe this sign. Notably, skin pinch may go back quickly in a severely malnourished patient such as a child with kwashiorkor even at dehydration condition. In such situation, monitoring the patient's weight is recommended to confirm dehydration*.

3 *Major signs*.

4 *For treatment of severely malnourished patients: ORS for moderate dehydration with no signs of shock (20ml/kg in first 2 hours at rate of 5ml/kg every 30 minutes, followed by 50ml/kg at rate of 5ml/kg/hour for up to 10 hours); and IV for severe dehydration with signs of shock (Ringer Lactate 15ml/kg/hour over 2 hours, followed by ORS 10ml/kg/hour until dehydration is corrected). Breast-feeding and therapeutic milk possible during oral rehydration*.

5 *Antibiotics (per EHNRI/EPHI guideline): For only severely dehydrated cholera patients; after IV rehydration. Mass chemoprophylaxis not recommended for cholera outbreak control. Selective chemoprophylaxis (1 dose of Doxycycline) may be useful for household members sharing food and shelter with cholera patient. Doxycycline (1 dose): for adults (except pregnant women); contra-indicated in pregnant or breast-feeding women and children under 8 years of age but can be used to treat cholera as 1 dose should not have any adverse effects. Amoxicillin syrup: for children; can be used also for adults if other antibiotics not available or V. cholerae resistant to those. Erythromycin: for pregnant women; may be used if other antibiotics not available or V. cholerae resistant to those*.

### Transmission Control

The EPHI (EHNRI) guideline (20) notes disinfection of transport and houses of cholera patients as soon as cases are confirmed at health facilities. Chlorine solutions are to be used for house spraying. Household visits are also a good way to engage local populations on hygiene promotion and active case detection at community level. Distribution of water purification chemical to affected kebeles is recommended when cholera cases are reported. In shortage of water treatment supplies, it is recommended that resources are prioritized based on needs and high risks such as areas with: low latrine coverage, bordering affected kebeles, sharing common water sources with affected kebeles, located downstream from affected kebeles, camps with unsafe drinking water sources such as refugee camps, miliary, etc (20). As part of the cholera outbreak control at affected communities, local residents need to be informed of the epidemic and measures in place, emphasizing the need for early case identification, immediate referral to CTC/CTU/ORPs, and free-of-charge treatment of cholera cases (20). Communication guideline in cholera epidemics includes preventive WaSH measures at household and community levels and addressing misinformation and rumors. Several studies on past cholera outbreaks in Ethiopia highlighted the importance of prompt and effective response at the community level, involving community leaders and community-based health workers (22). Contaminated holy water sources have been often identified as one of the risk factors of cholera outbreaks in parts of the country (23). Community sensitization on behavioural change associated with WaSH such as proper hand-washing using soap, cooking of raw vegetables, consumption of safe and clean water are some of the important measures that individuals can take for protection against *V. cholerae* infection and transmission.

### Use of Oral Cholera Vaccine (OCV)

Based on the World Health Assembly (WHA) Resolution 64.15 adopted in May 2011, which emphasized an integrated and comprehensive approach to cholera control including the use of OCV, the WHO in consultation with technical partners has established an OCV emergency stockpile and its implementation framework in 2013 (24, 25). Vaccines for the stockpile are procured from WHO pre-qualified manufacturers at negotiated prices. The emergency stockpile is managed by the OCV ICG for vaccine provision, based on review of the ICG request form and a reactive vaccination plan submitted by respective countries affected by ongoing cholera epidemics (25). Currently the OCV stockpile is also used in non-emergency settings for cholera prevention in cholera hotspots in endemic areas under the provision of the WHO GTFCC OCV Working Group (WG) (26, 27).

The use of OCV has increased significantly in Ethiopia since 2019 as part of the government's cholera outbreak control measures. The OCVs can be used as a reactive vaccination campaign in cholera outbreak settings or pre-emptively in cholera high-risk endemic areas to prevent potential outbreaks. With the recent cholera epidemics affecting Ethiopia with over 56,000 cases during 2015–2021 from all regions across the country, the government of Ethiopia has approached the ICG and the government of the Republic of Korea via bilateral diplomatic channel in 2019 for OCV doses to conduct reactive vaccination campaigns (28). In response to the cholera outbreaks in 2019 and 2020, the Ethiopian government has requested over 4 million doses of OCV to WHO for two rounds of vaccination campaign in 22 cholera outbreak woredas in 2020 (29). Further in 2021, around 6.8 million doses of OCV were requested and approved by the GTFCC OCV WG for use in 29 cholera hotspots in Ethiopia based on the NCP put together by the Ethiopian government (30).

## Section 3: National Commitment for Cholera Control in Ethiopia

Overall, the government of Ethiopia has implemented the multi-year *Health Sector Development Plan (HSDPs)* since 1997 over the last 20 years (31–34). The Ministry of Health (MOH) has also developed and implemented the *Health Sector Transformation Plan I (HSTP-I) 2015/16-2019-20 (2008 Ethiopia Fiscal Year (EFY)-2012 EFY)*, and subsequently launched in 2020 a Joint Assessment of the National Health Strategy (JANS) to review the next phase *Health Sector Transformation Plan II (HSTP II) 2020/21-2024/25* (35). Overall, the HSTP-I has contributed to reducing morbidity and mortality of major communicable diseases in Ethiopia such as HIV, tuberculosis and malaria, but maternal and child health associated with infectious diseases still remain high (35). Further challenges in the health system in Ethiopia include high disparity in healthcare service utilisation and health outcomes among people at different geographical areas and socio-economic levels (35). Reflecting the outcome of HSTP-I, the next 5-year HSTP-II, aligned with national 10-year development plan, aims to accelerate universal health coverage (UHC), protect people from health emergencies, create Woreda transformation, make health system respond to people's needs (35). The HSTP-II includes cholera as one of the regular disease outbreaks caused by cyclical hazards along with measles and yellow fever (35).

The national guidelines specific to infectious diseases and public health emergency management in Ethiopia include the *Public Health Emergency Management (PHEM): Guidelines for Ethiopia*, EPHI (EHNRI), 2012 (36). This PHEM Guideline encompassed the International Health Regulations (IHR 2005, third edition; amendment adopted by the 67^th^ WHA in 2014 and entered into force for all States Parties in 2016) (37) that the government of Ethiopia has also ratified. Specific to cholera control, the EPHI (EHNRI) *Guideline on Cholera Outbreak Management Ethiopia* has been developed and available since 2011 (20), providing guideline on cholera control such as: cholera surveillance (case detection and notification), outbreak investigation, outbreak response (Epidemic Committees, roles and responsibilities of various stakeholders, case management, transmission control), cholera treatment centers (guidelines on establishing and managing CTCs), WaSH, communication, monitoring and evaluation, and cholera preparedness.

Further, the Ethiopian government has developed a multi-sectoral multi-year NCP (18) and submitted to the WHO GTFCC for Independent Review Panel (IRP) (38) in 2021. The Ethiopia NCP (Multi-Sectoral Cholera Elimination Plan in Ethiopia 2021–2028) has six main targets: (i) effective leadership and multi-sectoral coordination for cholera elimination; (ii) strengthened surveillance and laboratory capacity (laboratory culture and rapid diagnostic tests, assessment of antibiotic susceptibility of bacteria and tracking strains) at all levels for early case detection and case confirmation by 2028; (iii) cholera mortality reduction by 100% in hotspot woredas by 2028 and no local transmission in hotspot woredas; (iv) OCV vaccination with 97% coverage in hotspots (preventive) and in outbreaks (reactive); (v) increased basic water supply from 65 to 90% and sanitation and hygiene practice coverage from 6 to 80% by 2028; (vi) behavioural change in hotspot woreda population to contribute to reduction of cholera deaths by 100% (18). The hotspot analysis was conducted based on disease prevalence and persistence using the cholera hotspot mapping tool developed by the GTFCC with plans for annual revision (18).

## Section 4: Discussions on Challenges and Way Forward

The multi-year health sector development plans aligned with the national development plan exhibit the strong political commitment and leadership of the Ethiopian government for public health system strengthening and infectious disease management including cholera control. The NCP has been prepared by multiple government branches, including MOH, EPHI, PHEM Center, Disease and Health Surveillance and Response Directorate, Bacterial Disease surveillance and Response Case Team, Ministry of Water, Irrigation, and Electricity, National Disaster Risk Management Commission, Ethiopian Pharmaceuticals Supply Agency, Ethiopian Food and Drug Administration Authority, Regional Health bureaus, etc. in collaboration with Cholera Technical Working Group (CTWG) (18) The CTWG is composed of EPHI Public Health Emergency Management and Directorates of Infectious and Non-infectious Diseases Research, MOH Health Promotion and Disease Prevention General Directorate, as well as WHO and other external partners (18) Reflecting the WHO GTFCC guiding document on NCP development (39), the Ethiopia NCP has six pillars; leadership and coordination, WaSH, surveillance and reporting, OCV use, healthcare system strengthening, and community engagement (18). The high-level leadership from the Ethiopian government involving the Minister of Health and the Office of the Deputy Prime Minister has been instrumental in putting cholera on the national health agenda in Ethiopia and demonstrating government commitment.

In order to achieve the NCP goals in Ethiopia, improved disease surveillance, diagnostics capacity, health information reporting system, effective OCV intervention strategy, community engagement for early case detection and proper case management, and WaSH promotion is critical. Limited rapid diagnostics or laboratory diagnostics capacity particularly in remote and distanced areas from the capital and regional cities prohibits the early detection and accurate diagnostics of *V. cholerae* and other causative pathogens associated with diarrheal diseases, which may also lead to inappropriate use of antibiotics. As a result, available government records of cholera cases are often based on clinical diagnosis of suspected cholera. Improved capacity of systematic and quality surveillance and data recording in health system is needed. There are also gaps in the quality of routine health information system data in public health facilities at regional level compared to national level (40). Lack of trained personnel compromise quality of surveillance and data reporting. Regular training programs for health workers at regional, woreda, and kebele levels with committed supervision and feedback are essential (40). To enhance surveillance and laboratory capacity at all levels for early detection and case confirmation by 2028 and reducing cholera-associated mortality by 100% in cholera hotspot woredas by 2028 with no local transmission (18), sufficient structured capacity building program for health system strengthening is warranted. Further, investment in genomic surveillance and bioinformatics analysis capacity in-country will be an additional valuable asset, enabling the local public health officials and researchers to monitor the evolution and spread of *V. cholerae* and other infectious disease agents detected in Ethiopia.

Strengthened surveillance and quality reporting system will further allow the government to assess and evaluate the impact and effectiveness of vaccination when OCVs are used pre-emptively in cholera endemic hotspots or reactively in cholera outbreak settings. Improvement in the quality of population demographic census data will also contribute to better estimation of disease incidence, prevalence, mortality rates, and also vaccination coverage rates that can be one of the important variables to assess direct and indirect herd effect of OCV vaccination in cholera control. A recent review on the national Health Management Information System (HMIS) in Ethiopia exhibited discrepancies on some health indicators such as population data estimates and vaccine coverages on Ethiopia Demographic and Health Survey (EDHS) records and routine HMIS data (41). Due to the limited resources, low- and middle-income countries (LMICs) including Ethiopia often rely on surveys to gather various health sector data when routine health information system is not adequately functioning. However, such surveys are intermittently performed and do not necessarily cover all areas, resulting to lack of district-level data for better health planning. More attention to the implementation of routine data gathering and quality HMIS is important to address this gap on health sector data, and evidence-based health program interventions based on the needs identified at the sub-national levels.

An adequate OCV vaccination strategy for different cholera outbreak contexts and active case management at the community level are important to effectively prevent potential outbreaks, control transmissions and reduce unreported deaths attributable to cholera. Several innovative OCV vaccination strategies have been introduced in different cholera epidemic and endemic countries such as the case-area targeted interventions (CATIs) (42), ring vaccination (43, 44), self-administration of the second dose of OCV (45), and integration of WaSH intervention delivery at health facilities with vaccination program (46). A recent systematic reviews and case studies on CATIs showed the approach used in 15 outbreaks in 12 countries, including Democratic Republic of Congo, Haiti, Yemen, and Zimbabwe. The analysis showed interventions varied with WaSH interventions more commonly implemented, and alert systems triggering interventions diverse from suspected cholera cases to culture confirmed cases (47). A modeling study on CATI recommended using OCV, antibiotics, and water treatment interventions at adequate radius around cases in cholera epidemic control (48). A geospatial analysis on implications of ring vaccination in Kathmandu Valley, Nepal suggested considering a ring vaccination strategy in large urban areas with recurrent seasonal outbreaks, whereby specific outbreak locations are not predictable (43). In remote resource-limited settings, a self-administration strategy for second-dose OCV delivery in urban Dhaka, Bangladesh (45) and hard-to-reach fishermen communities in Malawi (49) were demonstrated feasible. Based on the Ethiopia NCP endorsed, preemptive OCV vaccinations in endemic areas will be based on the cholera hotspot mapping. Further innovative intervention approaches in varying cholera endemic and epidemic environments in Ethiopia should be explored and their impact on cholera control evaluated. Proactive community engagement for active cholera case detection at community-level and immediate case referral for proper cholera case management is critical.

A multi-sectoral approach requires an investment in improved availability and accessibility of WaSH infrastructure nation-wide and its adequate utilisation. Currently, the national sanitation coverage in Ethiopia reached around 57%, which amounts to more than 45 million people without access to appropriate sanitation facilities (50). The water supply and latrine coverage is particularly lower among households in lower socio-economic levels and in remote areas, as well as some large crowd gathering public sites such as market places, bus stations, religious gathering sites, and even schools that can be a potential cholera transmission hotspot (18). Only around 27% water supply coverage and 35% sanitation coverage is assessed in 45 woredas that have been identified as cholera hotspots (18). There are several WaSH projects in these cholera hotspot woredas such as One-WaSH, Co-WaSH, Woreda Transformation, humanitarian WaSH cluster activities, supported by external partners such as World Bank, United Nations Children's Fund (UNICEF), United Nations Office for the Coordination of Humanitarian Affairs (OCHA), bilateral government donors, and other non-governmental organizations (NGOs).^18^ The WaSH pillar constitutes nearly 55% of USD404 million, the total budget for implementing the Ethiopian NCP in the next 8 years (18) The transparent and efficient management of resources, including allocation and use of available resources, should be ensured by the Ethiopia government-led coordination of health sector for aid effectiveness. Accountable monitoring of financing and tracking of indicators are important for the success of national cholera control and other infectious disease control and prevention broadly.

Going forward, a comprehensive monitoring of the actual practice related to cholera case detection, healthcare facility and laboratory capacities on cholera surveillance, diagnostics and reporting, tracking of OCV usage and various WaSH projects, in comparison to the available national guidelines, will provide a more robust baseline to track NCP progress and assess impact of interventions. Lessons learnt from NCP development and roll-out in Ethiopia may serve as a reference for countries with similar public health agenda. Managing cross-border transmission of cholera and other infectious diseases especially with neighboring countries that share common water sources, corridors of transportation, and frequent movement of people remains an important area for multi-stakeholder policy dialogues and joint health research. Climate change may further pose past trends of cholera seasonality less predictable, which may lead to cholera and other infectious disease outbreak response and preparedness more challenging. Further research and public health interventions to address the knowledge and health service gaps concerning cholera in Ethiopia, as well as the trend and impact of climate change and other risk factors associated with infectious diseases are warranted. A systematic and robust monitoring and evaluation program is essential for the successful execution of multi-sectoral NCP roll-out in Ethiopia. Capacity building in cholera surveillance and laboratory diagnostics, health information reporting system, WaSH access and utilisation, early case detection and case management, effective OCV vaccination strategies, and community awareness on disease prevention at all levels in Ethiopia should be guaranteed.

## Data Availability Statement

The original contributions presented in the study are included in the article/supplementary material, further inquiries can be directed to the corresponding author/s.

## Author Contributions

SP: conceptualization and original writing. SP, YJ, SK, AG, DH, BY, ME, MG, and MT: review and editing. All authors contributed to the article and approved the submitted version.

## Funding

Publication fee is sponsored by LG Electronics and Korea Support Committee of the International Vaccine Institute (Grant Code: CHMTD05083).

## Conflict of Interest

The authors declare that the research was conducted in the absence of any commercial or financial relationships that could be construed as a potential conflict of interest.

## Publisher's Note

All claims expressed in this article are solely those of the authors and do not necessarily represent those of their affiliated organizations, or those of the publisher, the editors and the reviewers. Any product that may be evaluated in this article, or claim that may be made by its manufacturer, is not guaranteed or endorsed by the publisher.
